# Social media and moral panics: Assessing the effects of technological change on societal reaction

**DOI:** 10.1177/1367877920912257

**Published:** 2020-03-28

**Authors:** James P Walsh

**Affiliations:** University of Ontario Institute of Technology, Canada

**Keywords:** digital technologies, folk devils, moral panic, public communication, social media, social problems construction, societal reaction

## Abstract

Answering calls for deeper consideration of the relationship between moral panics and emergent media systems, this exploratory article assesses the effects of social media – web-based venues that enable and encourage the production and exchange of user-generated content. Contra claims of their empowering and deflationary consequences, it finds that, on balance, recent technological transformations unleash and intensify collective alarm. Whether generating fear about social change, sharpening social distance, or offering new opportunities for vilifying outsiders, distorting communications, manipulating public opinion, and mobilizing embittered individuals, digital platforms and communications constitute significant targets, facilitators, and instruments of panic production. The conceptual implications of these findings are considered.

Popularized in Cohen’s study of youthful hooliganism in post-war Britain, ‘moral panic’ constitutes a keyword in social-scientific studies of crime, deviance, and control. Referring to episodes where folk devils – moral outlaws and ‘visible reminders of what we should not be’ (Cohen, 2002 [1972]: 2) – are blamed for societal malaise, the term captures how ‘right-thinking’ actors transmute deviant outsiders into potent sources of anxious indignation. For many, the framework’s leading contribution was illuminating the media’s significant role in constructing and amplifying social problems (Critcher, 2003; Jewkes, 2015; Kidd-Hewitt and Osborne, 1995). For Cohen and his peers, panics represented media events, with journalists and broadcasters playing an essential role in identifying aberrant behaviour and mobilizing consensus and concern.

While the concept’s influence endures, seismic shifts in media space – the rise of social media and digital platforms – broaden access to information and are transforming the production of public knowledge. Given panics’ status as struggles over the boundaries of order, truth, and normality, such developments ‘pose some of the most interesting contemporary questions for moral panic theory’ (Falkof, 2018: 4). Reflecting appeals from several of the framework’s leading proponents (Critcher, 2017; Goode and Ben-Yehuda, 2010; Hay and Hall, 2013), Falkof (2018: 4–5) has argued that, to retain moral panic’s conceptual utility, scholars must interrogate how social media are ‘upend[ing] traditional . . . flows of information and power’. Despite repeated calls for deeper engagement with technological changes (Critcher, 2017; Mawby and Gisby, 2009), existing work has either remained silent or under-represented their diverse effects. Concerning the former outcome, in his recent research note, Hier (2018: 9) claims that moral panic scholars have neglected digital communications and continue to privilege mass-broadcasting in their analyses. When technological change is discussed, received accounts are partial and incomplete. Informed by McRobbie and Thornton’s (1995) influential discussion of ‘multi-mediated social worlds’, it is held that the fragmentation and multiplication of media systems – dynamics accelerated by ubiquitous online platforms – are diversifying information about public issues and broadening claims-making capacities, outcomes which empower alternative voices and render Cohen’s framework ‘inaccurate and unuseful’ (Krinsky, 2013: 9; cf. Goode and Ben-Yehuda, 2010: 98–100).

When viewed from the standpoint of the present, both perspectives contain significant omissions. One the one hand, eliding socio-technical changes promotes a static, universal model that smooths out complexity and encourages intellectual inertia. Conversely, critiques of Cohen’s framework neglect its continued relevance and are incommensurate with contemporary realities. Evinced in an upsurge of authoritarian populism that hinges on the scapegoating of despised others, affluent societies appear wracked by unremitting fear and resentment (Tiffen, 2019; Wright, 2017), conditions that, as argued below, are inseparable from changes in media. Accordingly, rather than jettisoning the moral panic concept or subjecting it to ‘ritualistic reproductions’ (Kidd-Hewitt and Osborne, 1995: 4), Cohen’s framework should be refined to consider how digital communications shape reactions and are appropriated to incite alarm.

Reflecting such concerns, this article surveys social media’s effects on the issues and claims-making patterns that propel moral panics. It argues that, whether generating anxiety about social change, sharpening social distance, or offering new opportunities for vilifying outsiders, distorting communications, manipulating public opinion, and mobilizing embittered individuals, digital platforms and communications constitute significant targets, facilitators, and instruments of panic production. Accentuating such dynamics is not intended to dismiss received perspectives but to invigorate them, promoting more holistic consideration of the implications of new media. To be sure, a handful of works have provided commentary on or case studies of social media and cognate technologies (for example, see Flores-Yeffal et al., 2019; Hier, 2018; Marwick, 2008; Wright, 2017). Despite their insight and contributions, knowledge of social media’s diverse effects remains scattered and fragmentary. Thus, while some of this article’s propositions can be gleaned from existing studies, it offers a systematic elaboration that aims to promote analytic balance and encourage productive exchanges that can orient future scholarship.

After revisiting the media–moral panic relationship, this article assesses how social media escalate the frequency and intensity of overwrought reactions. While addressing several concrete examples, particularly the role of digital communications in promoting extremist agendas, as recent events concerning Trumpism, Brexit, the alt-right, and ‘fake news’ have shattered myths regarding their positive and empowering qualities, the focus of this article is more on general claims than particular findings. Accordingly, rather than a final, definitive statement, it presents developmental suggestions and a heuristic that can, and should, be subjected to further scrutiny and debate. In the end, such preliminary efforts are significant as ‘before we can pose questions of explanation, we must be aware of the character of the phenomenon we wish to explain’ (Smelser, 1963: 5).

## Changes in media space: the rise of ‘multi-mediated social worlds’

While the identification and policing of deviance are perennial features of human groups, moral panics are ‘unthinkable without the media’ and are distinctive to modern, mass societies (Critcher, 2003: 131). In many respects, Cohen and his contemporaries (Cohen and Young, 1973; Hall et al., 1978; Pearson, 1983) were the first to articulate the essential role of news-making in constructing social problems. Beyond generating surplus visibility and making otherwise marginal behaviours appear pernicious and pervasive, the media represent an independent voice (Goode and Ben-Yehuda, 2010). By delineating moral boundaries and circulating dire predictions about monstrous others, the histrionic tenor of reporting sensitizes audiences, culminating in hardened sentiment and unbridled punitiveness (Wright, 2015). Moreover, coverage translates ‘stereotypes into actuality’, elevating the actual and perceived severity of deviance (Young, 1971: 11). Here, identifying affronts to moral order triggers virulent hostility, further marginalizing folk devils and amplifying their deviant attachments and identities. As a control culture is institutionalized, surveillance and intervention intensify, exposing additional deviance, confirming popular stereotypes and justifying further crackdowns (Garland, 2008).

Since Cohen’s research nearly a half-century ago, media systems have undergone sweeping transformation, leading many to question the continued relevance of his work. A particularly influential critique in these regards comes from McRobbie and Thornton (1995). For them (1995: 560), Cohen’s emphasis on mass-broadcasting and its social and institutional correlates – a univocal press, hierarchical information flows, monolithic audiences – is untenable in the context of ‘multi-mediated social worlds’.^1^ Specifically, it is held that the proliferation of media sources encourages exposure to alternative, if not dissenting, claims and reactions, ensuring that ‘hard and fast boundaries between ‘normal’ and ‘deviant’ are less common’ (McRobbie and Thornton, 1995; 572–3; cf. Tiffen, 2019). Moreover, expanded access to media technologies – portable camcorders, personal computers, editing software and so one – broadens the remit of expression, giving rise to media sources inflected with the interests of marginalized groups (Coleman and Ross, 2010). Able to ‘produce their own media’ and defended by ‘niche and micro-media’ (McRobbie and Thornton, 1995: 568), folk devils are no longer powerless victims and can ‘fight back’ (McRobbie, 1994; cf. deYoung, 2013; Thornton, 1995). Consequently, deviant outsiders and their supporters display greater capacity to contest and short-circuit panicked reactions, outcomes that render the success of moral crusades ‘much less certain’ (McRobbie and Thornton, 1995: 573).

Focused on the diversification of conventional media space, McRobbie and Thornton conducted their stock-taking precisely as media systems were being further destabilized. With the onset of the 21st century, digital platforms not only underpin but also constitute social life in affluent societies, with individuals’ identities and relations at least partly cultivated through computing infrastructures (Lupton, 2018). Among the most significant manifestations of ‘digital societies’ are social media. Whether as social networking (Facebook), micro-blogging (Twitter), or photo – (Instagram) and video-sharing (YouTube) sites, social media have profoundly reconfigured the production and exchange of information. As ‘many-to-many’ systems of communication, they promote vernacular discourse and creativity, permitting ordinary users to produce and distribute staggering quantities of ‘user-generated content’ (Keane, 2013; Yar, 2014).^2^ Digital platforms are also displacing the mass media as an information source.^3^ Finally, as loosely coupled networks of users, their structure not only promotes virality – the rapid and unpredictable diffusion of content – but also fosters an expansive virtual sociality (Baym, 2015; boyd, 2010). Here, various attributes – ‘likes’, ‘retweets’, hashtags (#), mentions (@) and so on – index and anchor communications, promoting awareness of others and uniting spatially dispersed users into communities of shared interest and identity (Murthy, 2013).

While McRobbie and Thornton could not have anticipated these momentous shifts, contemporary scholarship assumes, either overtly or implicitly, that their corrective remains as, if not more, relevant today (for example, see Carlson, 2015; Carrabine, 2008; Fischel, 2016; Marres, 2017). With information control representing a critical axis of power, social media are frequently depicted as an elite-challenging ‘microphone for the masses’ (Murthy, 2011; cf. Gerbaudo, 2018; Jenkins, 2006. Here, the accessibility and sophistication of digital platforms is believed to empower ordinary citizens to make their own news, name issues as public concerns, and shape collective sentiment (Coleman and Ross, 2010; Turner, 2010). With knowledge production and image-making increasingly steered by non-experts, many perceive citizen journalism as breeding accounts of reality rooted in public-mindedness rather than sensationalism or commercial considerations (Goode, 2009). In light of such developments, noted panic scholars claim digital media are shifting ‘the locus of definitional power’, ensuring ‘more voices are heard’ (Critcher, 2017) and generating ‘new possibilities for resistance’ (Lindgren, 2013: 1243). Thus, the increasingly nodal configuration of media space has attenuated moral guardians’ influence, ensuring that panics are ‘more likely to be blunted and scattered among competing narratives’ (Goode and Ben-Yehuda, 2010: 99; cf. le Grand, 2016).

## Moral panics and new media: reconsidering the relationship

While McRobbie and Thornton’s claims remain influential, their ability to convincingly order the evidence is considerably more limited than recent analysis suggests. In accentuating social media’s progressive consequences – information pluralism and robust opportunities for citizens to access the public sphere and defuse frenzied reactions – existing scholarship neglects how digital platforms are ‘underdetermined’ and double-edged (Monahan, 2010). Informed by such issues, the following offers a counterpoint, detailing how social media’s affordances intensify the proclivity to panic. Whether as objects of unease, sources of acrimonious division, or venues for staging moral contests, on balance, contemporary media systems promote febrile anxiety.

## Social media as an object of anxiety

Changing communicative and informational conditions frequently incite moral restiveness. As Cohen himself intimates (2002 [1972]: xvii), societies are regularly gripped by fears that, if improperly governed, new media will have deleterious effects on younger generations. The latest iteration of so-called ‘media’ (Drotner, 1999) or ‘techno-panics’ (Marwick, 2008), reactions to social media encapsulate deep-seated anxieties about social change and the types of people it begets.

Like prior episodes involving ‘dangerous’ media, including ‘penny dreadfuls’, pinball machines, comic books and ‘video nasties’, youth are ambivalently constructed as threatened and threatening (Springhall, 1998). While anxieties have surfaced around vulnerability stemming from, *inter alia*, online predators, sexting, cyber-bullying, and exposure to violent and pornographic content (Barak, 2005; Gabriel, 2014; Lynch, 2002; Milosevic, 2015), youth are also positioned as undisciplined and pathological, with social media branded a leading culprit. Alongside being blamed for moral failings – obesity, addiction, disengagement, cultural vacuity, solipsism (Baym, 2015; Thurlow, 2006; Szablewicz, 2010) – multi-media platforms have been linked to violent criminality. Whether in relation to video game violence, the possibility of obtaining information about weaponry and prior incidents, or the promise of celebrity immortality offered through documenting their grievances and attacks, digital media have been maligned for encouraging school shootings and associated massacres (Ruddock, 2013; Sternheimer, 2014).^4^ Further, during the 2011 England riots, journalists and politicians referenced BlackBerry and Twitter ‘mobs’, claiming teenage gangs employed digital communications to evade authorities, publicize lawlessness and coordinate anti-social behaviour (Crump, 2011; Fuchs, 2013).

Such fears have frequently culminated in attempts by adult society to intensify surveillance, censorship, and control over online platforms. For such crusaders, who often utilize the very technologies they condemn to whip up outrage, techno-panics provide an alibi for manning the ‘moral barricades’ and reasserting the hegemony of their values (Sternheimer, 2014). Thus, while they may empower grassroots actors and disturb social hierarchies, technological changes equally engender moral backlash and nostalgia.

## Social media as a facilitator of division and hostility

Social media also reconfigure the external environment wherein panics occur. Frequently valorized for encouraging connectedness and encounters with diverse others, upon closer inspection digital platforms exert centripetal force, producing ‘filter bubbles’ (Pariser, 2011) and ‘information silos’ (McIntyre, 2018) which narrow social horizons and increase the likelihood of engaging with affective, and often acerbic, content. As news is increasingly digitally mediated, such dynamics reveal, *pace* McRobbie and Thornton (1995), there is no one-to-one correspondence between media and message pluralism.

Able to curate content at the expense of professional gatekeepers, social media allow users to construct information ecologies that are personalized and restricted (Sunstein, 2018). Such outcomes are exacerbated by social media’s ‘aggregative functionalities’ (Gerbaudo, 2018): the use of promotional algorithms to deliver tailored content (Rogers, 2013). For example, by assessing the volume of ‘clicks’ (likes, shares, mentions, etc.) communications receive, Facebook’s customized news feed determines what is worthy of users’ attention, filtering out stories deviating from extrapolations of their interests and preferences (McIntyre, 2018). As this and related examples suggest, by amplifying users’ biases and aversions, social media encourage confirmation bias and isomorphic social relations (Powers, 2017).

Social media also favour content likely to generate significant emotion and outrage. By promoting communications based on predicted popularity, they prioritize and reward virality and the intensity of reaction rather than veracity or the public interest (Van Dijck, 2013; Yardi and boyd, 2010). The result is the proliferation of ‘click-bait’, deliberately sensationalized content that captivates through affective arousal (Vaidhyanathan, 2018). More significantly, new media systems privilege incendiary communications. Research suggests that, even for the most staid users, the *frisson* of disgust is too alluring as content unleashing fear and anger about out-groups is considerably more likely to garner attention and ‘trend’ (Berger and Milkman, 2012; Vosoughi et al., 2018).^5^ These dynamics ultimately appear contagious as messages’ emotional valence ‘infects’ other users, influencing their subsequent interactions and escalating bitterness and antipathy within online environments (Kramer et al., 2014; Stark, 2018).

Together such conditions promote anxious alarm. By allowing users to remain cloistered within their preferred tribes and visions of reality, digital platforms encourage misrecognition and distort understanding of social issues, making the acceptance of bloated rhetoric more likely (Albright, 2017). Accordingly, they obstruct heterogeneous interactions and exposure to opposing perspectives, dynamics long identified as precluding the root causes of panics – intolerance and hostility (Murthy, 2013). Finally, by inflating the visibility of inflammatory content, social media mobilize animosity towards common enemies and transform uneasy concern into full-blown panic.

## Social media as instruments of panic production

Alongside breeding fissiparous societies, multi-media platforms can be wielded to engineer crises. Historically, panics require the mass media to generate sufficient concern and indignation. Social media expand the pathways of panic production. As detailed below, by allowing ordinary netizens to identify and sanction transgression, they unleash participatory, crowd-sourced panics. Additionally, as architectures of amplification, their structural features can be commandeered to promote moral contests that are surreptitious, automated, and finely calibrated in their transmission and targeting.

### Participatory panics

Conventional wisdom suggests that panics are spearheaded by seasoned and advantageously positioned activists and elites. By expanding capacities of media production and distribution, digital communications permit citizens to directly publicize issues and promote collective action. Typically this has been associated with amateur news-making and attempts to document injustice and promote transparency and accountability (Coleman and Ross, 2010; Walsh, 2019a; Yar, 2014), but scholars have recently documented opposing trends, where social media are appropriated to define and enforce public morality. As lay actors increasingly participate in the exposure and sanctioning of deviance, distinctions between the media, the public and moral entrepreneurs are blurring, ensuring that panics stem from unorthodox sources and display new discursive and interactional contours.

On the one hand, social media enable micro-crusades that, while lacking broad public appeal and support, are sustained by dispersed groups of devoted and technologically equipped citizens. Whether employed to advance claims that Harry Potter promotes Satanism and the occult to impressionable youth (Sternheimer, 2014) or discredit public officials and assert a link between vaccinations and autism (Erbschloe, 2018), digital environments offer optimal arenas for uniting the conspiratorial. Given their accessibility and ease-of-use, they obviate the need for elite participation, promoting patterns of mobilization around issues where all citizens potentially emerge as crusaders (Hier, 2018). Moreover, social media’s ‘mob-ocratic’ tendencies can activate collective effervescence (Gerbaudo, 2018), producing panics driven by mass collaboration. Falling into this category are online ‘firestorms’ (Johnen et al., 2018), spontaneous and electric outbursts where the documentation and exposure of moral breaches – petty theft, public outbursts, drug use, sexual promiscuity, etc. – are rapidly disseminated, igniting interactive cascades of denigration (Trottier, 2018; Wright, 2017). Such episodes often culminate in digital vigilantism: forms of extra-judicial punitiveness – ostracism, doxing, harassment, job loss, physical attacks, death threats – that emerge from below (Powell et al., 2018; Trottier, 2017).^6^ Consequently, alongside increasing the frequency and velocity of panics, online environments appear to promote heightened virulence and excoriation. While underpinned by emergent technologies, forms of digitally mediated opprobrium are inseparable from late-modern social conditions as they offer a palliative for ontological precarity and allow otherwise atomized individuals to police social boundaries (Ingraham and Reeves, 2016; cf. Bauman, 2013).

### Architectures of amplification

Beyond expanding the profile of moral entrepreneurs, the networked and digital configuration of social media can also be marshalled to distort information flows, promote incendiary content, and channel user experience and engagement. In such instances, digital platforms constitute architectures of amplification that allow interested parties to punch well above their weight.

#### ‘Attention hacking’ and media manipulation

On the one hand, digital platforms permit highly energized and sustained groups to sculpt public sentiment by maximizing the visibility of ‘information pollution’ and ‘fake news’ – arresting, sensational and morally tinged content designed to distort and agitate (Kalsnes, 2018). Whether by steering communications, creating fake accounts, or exploiting digital interactions, techniques of ‘attention hacking’ can strategically influence engagement patterns and produce wildly disproportionate effects (Marwick and Lewis, 2017). Ultimately, by allowing users to eliminate ambiguity and delineate moral boundaries in publicly visible ways, sites like Twitter and Facebook generate new types of agency that can rapidly propel the ideas and identities of various outsiders into prominence (Joosse, 2018).^7^

With their cacophonous character making it difficult to vet the integrity of content, digital platforms have been inundated with captivating, tendentious and skewed, if not entirely spurious, communications (news stories, videos, memes, blog posts, hashtags, etc.) to distort online conversations and mobilize receptive users. An exemplary case of digitally mediated crusades appeared during the 2016 American election as dedicated members of the ‘alt-right’, as well as digital mercenaries employed by the Internet Research Agency (IRA), a Russia-backed ‘troll farm’, devoted considerable energy and resources to shaping political communication and behaviour. Central to their efforts was the creation, sharing, liking and promotion of misinformation and provocative discourse about contentious sociocultural issues, including race relations, gun control, abortion, Islamophobia and men’s rights (Bradshaw and Howard, 2017; Nagle, 2017; Singer and Brooking, 2018).^8^ Armed with an appreciation of digital platforms’ value in shifting the parameters of public discourse, such actors succeeded in generating virality, obtaining mainstream press coverage, and inciting considerable outcry and anxiety (Phillips, 2018).

More recently, the role of digital communication in spreading fake news and inciting panic was on full display in initial reactions to the novel coronavirus (COVID-19), an infectious respiratory disease of zoonotic origin. Following its emergence in Wuhan, China in January 2020, widespread scapegoating and fear-mongering erupted across social media. In relation to the former, the virus was racialized, with numerous messages linking it to the ostensibly exotic dietary practices and unsanitary behaviour of Chinese populations, with representations depicting them as folk devils and dangerous, impure others (Yang, 2020).^9^ Reflecting a ‘politics of substitution’ (Jenkins, 1992), such claims-making diverted attention from considerably more deadly (and preventable) diseases (e.g. malaria), as well as, the structural conditions – media censorship, political corruption, weakly enforced health and safety standards – underlying the emergence and rapid spread of the disease. Digital platforms were also used to circulate misinformation and dire, if not apocalyptic, predictions with various rumours – whether false reports of positive cases and contaminated Chinese imports, stories of individuals absconding from quarantine zones, or claims that the virus was a bioweapon developed by the Chinese or American governments – outpacing official information during the early stages of the outbreak (Bogle, 2020). By contributing to a broader climate of suspicion, such communications appear to be reactivating fears of a ‘yellow peril’, as well as producing emergency measures (enhanced surveillance, quarantines, travel bans etc.) and everyday expressions of racism and anti-Chinese sentiment (Dingwall, 2020; Palmer, 2020; Yang, 2020). As this example reveals, like prior epidemics (SARS, AIDS, etc.) where media coverage promoted fear and opprobrium about various outsiders (gay men, drug users, foreigners; see Muzzatti, 2005; Ungar, 2013; Watney, 1997), digital communications also play a significant role in distorting understanding and encouraging over-reaction. The episode equally suggests, however, that social media’s anonymous, horizontal structure ensures that messages travel exponentially faster, lack clear origins and feature palpable vitriol, outcomes that escalate the impetus and excess of alarm (Miller, 2020).

The spread of information pollution frequently hinges on perceptions of social media as the embodiment of the *vox populi* (Gerbaudo, 2018). Here, fake accounts are utilized to raise awareness and bolster the credibility of favoured content. On the one hand, advances in artificial intelligence allow bots – machine-led communications tools that mimic human users and perform simple, structurally repetitive, tasks – to spread ‘computational propaganda’ (Bradshaw and Howard, 2017; Ferrara et al., 2016). As social machines and artificial voices, bots automate and accelerate diffusion and engagement, creating, liking, sharing, and following content at rates vastly surpassing human capabilities. Thus, they facilitate viral engineering; expanding the momentum of certain messages and, in the process, altering information flows. To exude authority and authenticity, content is also circulated by bogus, ‘sockpuppet’ accounts posing as those of accredited experts (scientists, journalists, etc.) or ordinary citizens belonging to various groups (women, blue-collar workers, police officers, urban youth, etc.) and appearing to possess folk wisdom (Bastos and Mercea, 2017; Marwick and Lewis, 2017). Whether manual or automated, techniques of media manipulation also control narratives by reducing the visibility of unwanted and objectionable content. Here, keywords and hashtags affiliated with opposing perspectives can be ‘hijacked’ as platforms are flooded with nonsense or negative messages to disrupt and drown out specific communications, denuding them of their salience and influence (Woolley and Howard, 2016).

A recent example of such efforts is found in Twitter communications concerning the intensity of the 2019–20 Australian bushfires, an outcome widely linked to the longer fire seasons produced by climate change. The preliminary results of research conducted by Graham and Keller (2020) suggests that, at the height of the crisis, a coordinated misinformation campaign was waged by a sprawling network of troll and bot accounts to advance broader narratives of climate denial. By flooding social media with hashtags like #arsonemergency (in place of #climateemergency) and co-opting those already trending (e.g. #australiafire, #bushfireaustralia), such actors sought to publicize conspiracies that criminal elements – whether arsonists, radical environmentalists, or ISIS fighters – were responsible for the blazes and that climate change is an elite-engineered hoax and form of population control (Knaus, 2020).

Finally, the propagation of misinformation involves attempts to harness social interaction and collective sense-making. Studies suggest that distorted, emotionally charged content is considerably more likely to be shared by ordinary users who unwittingly enlarge its sphere of influence (Albright, 2017; Tanz, 2017).^10^ By bearing the imprimatur of whomever shared it, whether a relative, colleague, neighbour, or opinion leader, the substance of messages is validated and appears authentic as it spreads laterally across users’ networks (van der Linden, 2017). For instance, on several occasions, accounts linked to the alt-right and Russian operatives have successfully ‘seeded’ content, goading journalists, bloggers, activists, and politicians (including President Trump) into endorsing particular communications and providing broader platforms (Phillips, 2018).

Since messages distributed through formal channels and hierarchical apparatuses are frequently perceived as self-serving and inauthentic, media manipulation provides a powerful vehicle of promotion. By engineering popularity and relevance, the discursive swarms unleashed by bots and fake accounts can generate an impression of credibility, unanimity and common sense, an outcome essential to normalizing particular modes of thought (Chen, 2015). Ultimately, by concealing the authors and agendas behind communications, such practices facilitate shadow crusades and astroturfing (Rubin, 2017).

While applicable to numerous topics, digitally mediated crusades are distinctly prominent in relation to issues – migration, crime and policing, or terrorism – identified as leading and recurrent sources of panic (Hall et al., 1978; Kidd-Hewitt and Osborne, 1995; Odartey-Wellington, 2009; Walsh, 2017, 2019c; Welch and Schuster, 2005), as well as, central topics in online conversations during critical political moments (Benkler et al., 2018; Evolvi, 2019). For instance, in their recent study of anti-immigrant crusades, Flores-Yeffal et al. (2019) observed how the indexing of social media communications through hashtags like #IllegalsAreCriminals and #WakeUpAmerica fostered networked discourses and connectedness, helping to construct scapegoats, circulate calls for action, and ensure that xenophobic rhetoric echoed throughout cyber-space (see also Morgan and Shaffer, 2017).^11^ Additionally, preceding the Brexit referendum, supporters of the far-right UK Independence Party utilized digital platforms to trigger and inflate fears about foreigners, circulating contentious claims about workforce competition, cultural displacement, crime and terrorist infiltration (Vaidhyanathan, 2018).

#### Computational crusades

Finally, social media unleash crusades that are data-driven, granular, and highly dynamic in their transmission and targeting. Here, the digital surveillance and marketing infrastructures that underpin social media’s profitability permit computational modelling of user data, promising greater awareness of audiences and encouraging claims-making practices involving extensive narrowcasting; behavioural and psychometric profiling; and the production of predictive knowledge.

While empowering users as participants and agents of communication, digital platforms also render them legible as vast tranches of information about their attributes (e.g. gender, race, income), activities (e.g. hobbies, movements, browsing habits), and associations (e.g. relational ties, organizational memberships) are continuously scrutinized for commercial, legal and political purposes (Nissenbaum, 2009). Once harvested, user data undergoes deep profiling, producing digital dossiers which sort individuals based on dozens, and potentially hundreds, of variables. Consequently, audiences are less collectivities to be influenced *en masse*, than individually calculable units, arrangements that permit those possessing the necessary resources and technological literacy to target users with highly customized messages (Zuboff, 2015).

Accompanying geodemographic criteria, algorithms can identify and calculate expressive energies and subjective orientations – moods, sensibilities, and emotions. With advances in machine learning and sentiment analysis, digital communications can be analysed to map meaning structures, and discern personality traits on scales previously unimaginable (Andrejevic, 2013; Stark, 2018). For example, Cambridge Analytica, a consulting firm hired to assist the Trump campaign’s online messaging, harvested data concerning online engagement for over 230 million Facebook users, pooling it with other information to develop a sprawling collection of psychographic profiles on potential voters and gauge their receptiveness to various messaging strategies (Vaidhyanathan, 2018). Heralding the rise of communications that, while reaching immense audiences, are highly differentiated, it is estimated that, with the assistance of big data analytics, Trump’s campaign disseminated over 6 million distinct online ads, with variations of individual messages, at times, surpassing 200,000 (Singer and Brooking, 2018).

Big data also yields inferential and predictive knowledge, with computer models unearthing correlations, extrapolating information about users, and forecasting reactions. Here, digital enclosures are mined to identify regularities against which users are continuously compared, outcomes that allow claims makers to anticipate content’s likely resonance and develop flexible outreach strategies (Baym, 2013).^12^ Practices of dataveillance are also recursive, as feedback in the form of engagement patterns is reflexively monitored to elaborate correlations and deepen knowledge of users (Neuman et al., 2014). Accordingly, digital communications double as iterative experiments where multiple messages can be distributed simultaneously to survey reactions and refine techniques of persuasion (Andrejevic, 2013).

In relation to panics, profiling user data liberates crusaders from ‘monolithic mass-appeal, broadcast approach[es]’ to issue mobilization (Tufekci, 2014). Rather than attracting support through unifying, ‘big tent’ issues, dataveillance facilitates agile micro-targeted crusades. Able to cleave populations into demographic and affective types, moral guardians can precisely ‘hail’ subjectivities, allowing them to combine mass transmission with individual connection and overcome what has traditionally been a Hobson’s choice between maximal exposure and intimate resonance. Consequently, moral contests promise to become exponentially more sophisticated, ensuring overwrought discourse reaches, motivates and energizes its intended targets. Moreover, given the expressive contours of panics, and the importance of emotions – anxiety, hostility, even hysteria – as levers of action (Walby and Spencer, 2012), the mining, measurement and classification of affective states allows crusaders to viscerally connect with audiences and strengthen their messaging.

## Discussion

As a distinct species of collective behaviour, moral panics represent contentious and intensely affective campaigns to police the parameters of public knowledge and morality. As such, they are necessarily dependent upon and constituted by claims-making, with interested parties historically seeking to actuate alarm by influencing the imagery and representations of the mainstream press, arrangements disrupted by recent upheavals in media space. To illuminate the complex relationship between panics and the broader socio-technical context in which they unfold, this article has surveyed the impact of digital communications, presenting a taxonomy of social media’s effects on the issues, conditions and practices that incite collective alarm. While displaying elite-challenging potential, social media are ultimately Janus-faced and contradictory. Alongside providing emergent sources of unease, they cultivate facilitating conditions and offer ideal venues for constructing social problems. Specifically, by elevating agitational discourse and promoting homophily, social media generate social friction and hostility. Moreover, as instruments of panic production, new technologies reshape the identification and construction of deviance, both permitting lay participation and allowing various parties to manipulate public communications in ways that produce outsized, imperceptible and highly efficient influence.

While gauging the precise effects of social media requires more rigorous scrutiny than can be provided here, the available evidence indicates that, all things considered, they inflate the incidence and severity of panics. On the one hand, various studies suggest that, as architectures of amplification, digital platforms reduce transaction costs and transform peripheral (as well as automated and artificial) voices into conspicuous claimants (Vaidhyanathan, 2018).^13^ They also appear to enhance the spread of information pollution, with scholarship revealing that, whether transmitted by algorithms or human agents, ‘misinformation, polarizing, and conspiratorial content’ (Howard et al., 2018: 1) not only ‘diffuse[s] significantly further, faster, [and] deeper’ on social media (Vosoughi et al., 2018: 1146; Albright, 2017) but also, during the final days of the 2016 election, represented the most popular informational content on Facebook, leading many to speculate that it played a decisive role in Trump’s victory (Waisbord, 2018). Finally, evidence surrounding the extent to which gross distortions, extremist views and readily falsifiable conspiracies (such as the views that: climate change is a manufactured crisis, violent crime is at historic highs, undocumented migrants are overwhelmingly violent criminals, etc.) are being normalized as public idiom gives considerable cause for concern (McIntyre, 2018; Scheufele and Krause, 2019).^14^

Beyond advancing understanding of the media–moral panic relationship, an important task in its own right, by initiating dialogue between theoretical expectations and empirical instances, the preceding analysis promotes conceptual refinement and renewal. Specifically, accounting for social media’s effects on panic production illuminates significant mutations surrounding the interactants, functions and communicative patterns that define contemporary crusades. First, as many-to-many systems of communication, social media promote novel patterns of participation, offering ordinary persons a greater role, facilitating spontaneous outbursts driven by multitudes and introducing automated, machine-led campaigns. Additionally, in enabling new techniques of media manipulation, digital platforms contribute to the weaponization of panics. While conventional wisdom suggests that panics represent domestic affairs, oriented towards mobilizing support, acquiring power and status or manufacturing consent, the case of Russia and information warfare suggests that normative conflict may be exogenously engineered to provoke significant social and psychological disruption. Finally, in place of uniform messages and mass appeal, the combination of data-mining and behavioural profiling unleashes claims-making techniques that are inhabited and hyper-targeted.

Drawing attention to these features exposes significant transformations and bolsters the versatility and explanatory capacity of Cohen’s paradigm. Thus, mirroring other recent interventions (Falkof, 2018; Joosse, 2018; Wright, 2017), by accounting for emergent social conditions, this article advances a nuanced, flexible framework rather than a fixed, uniform model. Ultimately, exposing anomalous findings that push the limits of existing perspectives extends the concept’s range of applicability, promoting a more robust framework capable of accommodating pivotal shifts in media space and the social relations they engender. Alongside laying the foundation for further empirical applications, given the depth and rapidity of social change, such conceptual dexterity is an asset rather than a liability (Goode and Ben-Yehuda, 2010; Jewkes, 2015).

## Conclusion

As an account of reaction and social problems construction, moral panic theory has traditionally emphasized the mass media’s role in sculpting collective knowledge, arbitrating between the real and represented, and generating significant discrepancy between risk and response. This article suggests that, while the legacy press continues to play a significant role, with the ubiquity of digital platforms and technologies, the emergence and spread of panics is being reconstituted. In particular, scholars can further refine and expand the concept’s range and impact by engaging with social media’s diverse and far-reaching effects on the contours of collective alarm. While it is admittedly premature to predict what new attributes media systems will assume, and there is too much contingency to suggest that future developments will follow an inexorable path, it is hoped that, by taking technological change into account, the idea of moral panic will continue to influence understandings of how fear and transgression are mobilized for varied purposes.

## Notes

1. In relation to Cohen’s work, McRobbie and Thornton’s (1995) critique continues to be cited as a core ‘dimension of dispute’ (David et al., 2011; cf. Carrabine, 2008; Garland, 2008; Goode and Ben-Yehuda, 2010).

2. For instance, Facebook’s 2.38 billion active users leave roughly 300,000 comments per minute and share over 5 billion posts per day (Chandler and Fuchs, 2019; cf. Walsh and O’Connor, 2019).

3. In 2017, two-thirds of American adults obtained some of their news from social media (Shearer and Gottfried, 2017), while, for British and North American youth, it represents their primary news source (Wakefield, 2016).

4. An exemplary case is Pekka-Eric Auvinen a Finnish shooter deemed the ‘YouTube gunman’ after using the video-sharing site to publicize his actions, espouse nihilistic views, and share a final message immediately before killing eight people (Lindgren, 2011; Walsh, 2019b).

5. One study of Facebook found the ‘click-through’ rate for socially divisive content exceeded typical ads by tenfold (Ribeiro et al., 2019).

6. In a striking example of online shaming and digitally mediated outrage, moral entrepreneurs associated with anti-paedophile activism in Canada, the UK, and Russia have all employed digital platforms to investigate, identify, expose and censure suspected sex offenders (Favarel-Garrigues, 2019; Trottier, 2017).

7. Citing Donald Trump’s rise as a charismatic political maverick, Joosse (2018) argues that non-traditional media are ideally suited for producing and reiterating simplistic and highly resonant moral categories, outcomes that can endow otherwise peripheral parties with significant power and influence.

8. While a full discussion exceeds the scope of this article, the alt-right encompasses an ill-defined amalgam of actors (white nationalists, men’s rights activists, palaeo-conservatives, nativists, etc.) united by opposition to ‘identity politics’, multiculturalism, and perceived ‘political correctness’ (Hawley, 2017; Nagle, 2017).

9. For instance, videos of Chinese citizens eating bats, rodents, snakes and other ‘dirty’ or ‘exotic’ wildlife were quickly posted and widely distributed across various social networking sites (Palmer, 2020).

10. Surveys from the USA reveal one-quarter of respondents have knowingly shared misinformation on social media (Barthel et al., 2016).

11. Russian operatives also contributed to such efforts, distributing content and even organizing protests through fake Twitter and Facebook accounts (Singer and Brooking, 2018).

12. Research reveals, for instance, that various attributes – sexuality, religiosity, education, etc. – can be reliably predicted from patterns involving the single data point, Facebook ‘likes’ (Markovikj et al., 2013).

13. For example, during the 2016 election, content from just six Russian-backed Facebook accounts garnered 340 million shares and nearly 20 million interactions on the platform (Matsakis, 2018). Additionally, whether deployed by foreign agents or domestic extremists, bots produced one-third of posts concerning the 2016 Brexit vote, despite representing just 1% of active Twitter accounts (Narayanan et al., 2017).

14. For instance, over two-thirds of Americans claim that fake news has left them disoriented and confused about basic facts (Barthel et al., 2016), while another survey revealed 75% of Americans familiar with a fake news headline thought it was accurate (Roozenbeek and Van der Linden, 2019).
